# Organotherapy

**Published:** 1912-08

**Authors:** Edward T. Smith

**Affiliations:** Buffalo, N. Y.


### Organotherapy

By EDWARD T. SMITH, M.D.
Buffalo, N. Y.

ORGANIC chemistry in the past ten or fifteen years has given to the medical profession some of its greatest remedies to combat disease and deserves more attention from the general practitioner.

What would medical history in that time have been but for the experiments and discoveries of Pasteur, Roux, Behring, Flexner and a host of others not so well known? It would be only a continuance of the old methods with different forms of drugs and combinations which medical science has been carrying on from the beginning of time and with the same uncertain and unsatisfactory results. Organic chemistry has robbed diphtheria of its terrors with antitoxin. Hydrophobia is successfully combated with the Pasteur serum. Adrenalin has proved its great superiority over all drugs in controlling certain forms of haemorrhage and saving life and Flexner has discovered a serum that robs the dreaded cerebro-spinal meningitis of its victim. The elaboration by Wright of bacterins has revolutionized the treatment of many diseases.

I do not include in the list of these newer remedies certain advertised combinations of animal extracts with iron, nux vomica, etc., nor the so-called "goat lymphs," lymphines and combinations of that character which are neither scientific nor useful.

It is unfortunate that in the past organic medicine was a wide field for quackery and the occult, but this day has gone by. In the light of modern science it is the mainstay of medicine and the hope of the human race in great epidemics; and the names of Brown-Sequard and Hammond (whose student I had the honor to be) will stand out prominently as among the great benefactors of mankind.

Extravagant claims have been made by certain manufacturers of animal extracts from the pancreas, spleen, ovaries, mammæ, kidneys and liver. I have used all their makes, and even those made in my own laboratory, under my own personal supervision, and I consider them inert, with one exception: the corpus luteum. This portion of the generative organs has demonstrated its value in women suffering from the many disagreeable symptoms of the change of life, controlling the hot flashes, palpitation of the heart, and all disagreeable symptoms of that period. It is the only remedy that has any value in this critical period of a woman's existence, and we owe it to organic chemistry.

I regard dessicated testes and cerebrum as inert, but I cannot say that in regard to the hypodermatic use of testes. A properly made orchitic extract prepared with glycerine and diluted with normal salt solution has very decided tonic properties in all degenerative diseases and the lymphatics prepared in the same manner give equally good results. A word of caution is here given never to apply any commercial animal extract to an abraded surface. If you will only moisten a small portion with sterile water and look at it through a ¼ inch objective you will get an object lesson in bacterial life.

It is not safe to inject an albuminous extract of the brain for several reasons. One reason is on account of the impossibility of making a perfectly sterile extract, and another is the danger from the neurin in the substance of the brain; any reaction that has ever been noticed from the injection of so-called brain extracts has, in my opinion, been due to the action of neurin upon the central nervous system. The brain of the sheep contains practically no neurin, and only in exceptional cases a very small portion of cholin, and should be used to make the extract for hypodermatic injection.

I can frankly say I never have been able to make an abuminous extract that I would inject. Poehl has prepared an extract of the brain for hypodermatic use which he calls opocerebrin, but for therapeutic results I class it with his preparation which he called spermin—an inert substance sold at a very high price.

There is no one alkaloid of any organ that will take the place of the organ itself; neither adrenalin, spermin, opocerebrin, nor others.

We now come to the one great draw-back of organotherapy and that is the hypodermatic needle. Patients dread the needle and as in some cases repeated injections are called for they refuse to continue treatment. I have given a great many thousands of hypodermatic injections and have no trouble and the reason is I always use a sharp needle, pinch up the flesh instead of spreading it as is usually advised, and never inject when I feel any obstruction. Never use force in injecting as it is better to withdraw and re-insert. It is not the injected matter that hurts but forcing a dull needle or one of too large calibre through the sensitive skin. In extra-sensitive persons you can spray the part with ethyl chloride. Use a sub-Q syringe and do not economize on needles as they are cheap and your glass syringe will prevent injecting air. By always injecting deeply into the connective tissue—never in the muscular—and using slight massage with alcohol afterwards, I have never had an abscess and only a very few nodosities which disappeared in a few days. After the first injection I have no trouble in inducing patients to continue treatment.

For the past few years I have been using empirically as a tonic and reconstructive in all degenerations and chronic infectious diseases, an extract made from bull's testes, mesenteric lymph glands and sheep's brain; forty per cent. each of brains and testes and twenty per cent. lymph glands, made by boiling the above in absolute alcohol, extracting the remaining proteids by succussion and evaporating the residue on a water bath. The result is a slightly brown crystalline mass; containing spermine from the testes, the two brain spermines, a lecithin from the lymph glands; and other extractives, the composition of which I do not know; the whole possessing all the tonic and special properties of the several organs. If a trituation of this extract is made with sugar of milk, and one minim of guaiacol added to each 2000 grains of mass it will not deteriorate for a long time. This small proportion of guaiacol must be added as some of those extractives are abumenoids and the mass will become mouldy in a short time if it is omitted. It can also be made in solution with 20 per cent. alcohol which will prevent change. Dixon first demonstrated that the nucleo-proteids and organic salts in the testes and brain were unaltered by boiling.

For hypodermatic use the extracted phosphorized fats can be used as a vehicle and it is advisable to put up each injection in a sealed anpoule if not used at once. I do not claim any particular virtue from the phosphorized fats but they make an ideal vehicle. If a preservative is used I prefer chloride of gold one to 5000 to trikresol.

The dosage is a very important matter and I generally give ½ grain of the extract triturated with sugar of milk three times daily, or the same amount in solution. The triturate tablet is very convenient. If used hypodermatically one ampoule daily will answer nearly all purposes, except in very serious cases. The effects of any overdosage are a sense of exhilaration and a rapid pulse. The pulse soon subsides but the stimulation of the senses persists for a long time and is followed by profuse diuresis. There is no actual danger from an overdose, but over stimulation should always be avoided.

I have used this form of extract for the past few years for internal administration in locomotor ataxia, nephritis, tuberculosis, asthma, hay fever and all degenerations and chronic infections, with the most satisfactory results, it being superior to any form of treatment laid down in the text books for these diseases.

I speak not only from my own experience but from that of many other physicians in all parts of the United States who have been supplied from my laboratory. It has been demonstrated that this combined organic extract is the nearest approach to a specific cure in asthma and hay fever that is before the medical profession at the present day.

It makes no difference how long standing the case may be or how complicated with other diseased conditions, the systemic tonication of this remedy acts better than any treatment laid down in the text books. Even in the great scourge of the human race, tuberculosis, it has proved its great superiority over all other remedies and I use it now to the exclusion of everything else. It is wonderful how it improves nutrition and innervation, and it never disagrees with the patient. All nauseous mixtures, oils, emulsions and cough mixtures are unnecessary with this extract and must be forbidden. The only restriction in diet is avoidance of too much starchy food, and excessive crowding of nourishment in tuberculosis. I cannot see the reasoning that makes physicians crowd in several times what a healthy stomach could digest, and if the digestive tract was in a healthy condition it would be unnecessary.

The first effect upon the digestive tract on giving the extract, is its local tonic action, and, secondly, an almost selective action upon the desquamative necrosis of the mucus membrane of the stomach and intestines. In incipient cases of tuberculosis the so-called pre-dyspepsia soon disappears, and in advanced cases the fermentation, distress and gas formation are soon controlled. In advanced cases with sweats, chills and fever, as soon as the stomach is in a healthy condition and digests food, and the restorative action of the digested food and the stimulant action of the extract begin to show their effects, these symptoms begin to abate. At the same time the distressing, hard cough from irritation of the vagus is ameliorated. The relief is remarkable; and no opiates are needed, which is providential, as they nullify any good action from the remedy.

The patient soon begins to gain in weight and strength and as these results are quick to show themselves you at once gain his confidence. All artificial emulsions of fats, tonics, containing iron, etc., are prohibited. There is no necessity for any other medication and none must be used.

It is always advisable to use the extract hypodermatically in conjunction with the internal administration of the extract, daily if possible, and, if not, as frequently as possible. By doing this you obtain better results than from using either alone and this is advised in serious and long standing cases. When the patient is very much emaciated it is always best to inject in the buttocks. In cases of long standing tuberculosis with extensive fibrous conditions no remedy can cure and the best that can be expected is relief and this can be more quickly obtained by this extract than by any other means.

I very strongly advise its use in all obscure cases of every character where there seems to be a want of tone or action in any special organ or organs in either sex. In premature senility it is particularly efficacious, clearing up the skin, removing wrinkled countenances and restoring elasticity to the skin. I use it in nervous diseases particularly in impotence for its beneficial action upon the spinal centers, injecting it supra-durally. Any physician with only a limited laboratory experience who can obtain the materials can make this extract and its mode of preparation by heat removes all danger of contamination. He will be repaid by the satisfactory results in treating many obstinate and obscure conditions which do not respond to the treatment laid down in the text books.

